# Anthropometric parameters of obesity can be alternative biomarkers for the potential cardiac dysfunction in obese children

**DOI:** 10.3389/fcvm.2022.850071

**Published:** 2022-08-18

**Authors:** Jing Sun, Li Wang, Yingjiong Lin, Yunfeng Liu, Fei Liu, Xumei Liu, Wenyan Dong, Wenqian Cai, Huimin Chen, Minhua Xiao, Hongfeng Luo, Xihong Liu, Jinzhu Duan

**Affiliations:** ^1^Department of Clinical Nutrition, Guangzhou Women’s and Children’s Medical Center, Guangzhou Medical University, Guangzhou, China; ^2^Department of Echocardiography, Heart Center, Guangzhou Women and Children’s Medical Center, Guangzhou Medical University, Guangzhou, China; ^3^Heart Center and Institute of Pediatrics, Guangzhou Women’s and Children’s Medical Center, Guangzhou Medical University, Guangzhou, China; ^4^Department of Laboratory, Guangzhou Women’s and Children’s Medical Center, Guangzhou Medical University, Guangzhou, China

**Keywords:** clinic investigation, cardiac dysfunction, anthropometric parameters, obese children, myocardial injury, blood lipid profile, cardiovascular disease

## Abstract

Childhood obesity, as one of the potential risk factors of cardiovascular diseases, is closely associated with the incidence of cardiovascular disease at a younger age and has become a public health concern worldwide. However, its potential effects on the cardiovascular system have still remained elusive. In this study, we systematically evaluated the cardiovascular characteristics of 79 obese children and 161 normal weight children in Guangzhou (China) using the potential biomarkers for cardiovascular disease. Compared with normal weight children, obese children not only exhibited significantly higher levels of creatine kinase (CK), lactate dehydrogenase (LHD), soluble fms-like tyrosine kinase-1 (s-Flt-1), high-sensitivity C-reactive protein (hs-CRP), and uric acid (UA) (*p* = 0.0062, 0.0012, 0.0013, 0.0225, and <0.0001, respectively) but also significantly higher diastolic blood pressure (*p* = 0.0074) and the heart rate (*p* = 0.0049) were found in obese children. Of 79 obese children, cardiac functions of 40 cases were further assessed by color Doppler echocardiography. The results showed that there were significant differences between the obesity group and the healthy weight group in terms of interventricular septal wall thickness at end-diastolic (IVSd), the left ventricular posterior wall thickness at end-diastolic (LVPWD), and aortic annulus (AO) (*p* < 0.0001, 0.0003, and *p* < 0.0001, respectively). Besides, the left and/or right ventricular functions were declined in 52.4% of obese children. Correlation analysis revealed that the anthropometric parameters of obesity were not only significantly correlated with a blood lipid profile but also exhibited a more significant correlation with most of the parameters of cardiac dysfunction than a blood lipid profile. Therefore, our study indicated that obese children in Guangzhou suffered from functional damages related to cardiovascular events, which were characterized by cardiac dysfunction, and the anthropometric parameters of obesity could be economically alternative biomarkers for monitoring of cardiac dysfunction in obese children.

## Introduction

Obesity is a serious public health concern worldwide. Childhood and adolescent obesity have reached epidemic levels in China (1). Various genetic, environmental, and hormonal factors contribute to obesity (2). Obesity can affect children’s functions, including their psychological and cardiovascular health, as well as their overall physical health. The association between obesity and other conditions indicates the necessity of study of obesity in children and adolescents (3).

Cardiovascular disease is a leading cause of morbidity and mortality worldwide. Obesity is one of the important potential risk factors of the cardiovascular disease. Due to the increase in the prevalence of obesity among children, several studies have been conducted to find out risk factors influencing the incidence of childhood obesity (4, 5). However, no strong correlation between childhood obesity and the characteristics of cardiac dysfunction has been reported.

In this study, after investigation of questionnaires, anthropometric parameters, body composition, and lipid indices, we concentrated on the cardiovascular-related indicators and cardiac dysfunction in children with obesity to determine the characteristics of cardiac dysfunction in obese children and to explore potential biomarkers for childhood obesity, enabling us to predict potential risk of cardiovascular disease in obese children.

## Materials and methods

### Study design and patients’ recruitment

The study protocol was approved by the Ethics Committee of Guangzhou Women’s and Children’s Medical Center (Guangzhou, China; Approval No. 375B00). In the present cross-sectional study, children with obesity were compared with healthy participants in terms of some cardiovascular-related indicators. Between September 2019 and December 2019, children who were admitted to Department of Clinical Nutrition of Guangzhou Women and Children’s Medical Center for obesity consultation were recruited. The inclusion criteria for the obesity group were as follows:

1. Body mass index (BMI) ≥ 95th percentile (6);
2. Children without the following diseases that cause obesity, such as Prader-Willi syndrome (PWS), Alstrom syndrome, Laurence-Moon-Biedl syndrome, etc., (6, 7);
3. Children aging 6–14 years old regardless of their gender or race, mainly including students in primary schools or middle schools;
4. Children without severe or acute obesity-related complications (e.g., acute heart failure, acute respiratory distress syndrome, sever hypertension, type 2 diabetes, etc.);
5. Children without primary or congenital metabolic disorders, such as urea cycle disorder, type 1 diabetes, etc.
6. Children without psychiatric disorders or organic encephalopathy, and those without history of receiving drugs for psychosis;
7. Children who did not receive drugs affecting nutrient absorption or metabolism in the recent 6 months (e.g., metformin hydrochloride, orlistat, atorvastatin, etc.);
8. Children who voluntarily participated in the study, and their parents approved their participation.

The inclusion criteria for the healthy weight group were as follows:

1. BMI > 3th and < 85th percentile;
2. Children aging 6–14 years old, regardless of their gender or race;
3. Children without primary or congenital metabolic disorders;
4. Children without psychiatric disorders or organic encephalopathy and those without history of receiving drugs for psychosis;
5. Children who did not receive drugs affecting nutrient absorption or metabolism in the recent 6 months;
6. Children who voluntarily participated in the study, and their parents approved their participation.

All children’s parents had signed the informed consent forms prior to enrollment.

### Data collection

The 24-h dietary intake survey was performed for 3 consecutive days, and the intake of carbohydrates, protein, fat, and total energy was calculated, which was then compared with the age-and sex-matched normal children [i.e., comparing with the energy and dietary intakes of children recommended in the Dietary Reference Intakes (DRIs) (8)].

For school children, their height and weight were measured by Seca704s electronic column scale, and their waist and hip circumferences were quantified by Seca201 band tape. One decimal was used for the results of height, waist circumference, and hip circumference, while two decimals were utilized for the results of weight. The measurement tools were regularly calibrated before utilization. All measurements were conducted by two trained nurses and compared with pediatric growth standards of the World Health Organization (WHO) presented in 2006 (9). The BMI was calculated as follows: BMI = body weight (kg)/height (m)^2^. The waist-to-hip ratio (WHR) was calculated as follows: WHR = waist circumference (cm)/hip circumference (cm). The waist-to-height ratio (WHtR) was calculated as follows: WHtR = waist circumference (cm)/height (cm). Sex- and age-adjusted BMI was compared with those of the WHO standards.

The body composition measurements were conducted by the InBody 720 system (Biospace Co., Ltd., Seoul, South Korea). Body composition, especially body fat percentage (BFP) and fat-free mass (FFM), was measured by the multi-frequency bioelectrical impedance analysis (10, 11).

Echocardiography was performed using a multi-purpose ultrasound machine (EPIQ7C; Philips Healthcare, Andover, MA, United States) by two trained and experienced pediatric echocardiographers. All children underwent the two-dimensional (2D)-guided M-mode echocardiography using a standard protocol, as described previously (12). The following cardiac measures were obtained over three cardiac cycles: aortic annulus (AO); the left atrial (LA) diameter; the left ventricular internal diameter at end-diastolic (LVIDd); the left ventricular internal diameter at end-systolic (LVIDs); interventricular septal wall thickness at end-diastolic (IVSd); left ventricular posterior wall thickness at end-diastolic (LVPWD); fractional shortening (FS%); the left ventricular ejection fraction (EF%); the left ventricular mass (LVM); the left ventricular myocardial mass (LVMM); tricuspid annular plane systolic excursion (TAPSE); and the right ventricular diameter (RVD).

### Statistical analysis

All data were analyzed using SPSS 25.0 software (IBM, Armonk, NY, United States). Continuous data were tested for normal distribution using the Kolmogorov-Smirnov test. Continuous variables with normal distribution were presented as mean ± standard division (SD), and were tested using the independent-samples *t*-test (intergroup comparisons) or repeated measures ANOVA and the Student–Newman–Keuls (SNK) *post-hoc* test (intergroup comparisons in time). Frequency (percentage) was used to describe the categorical variables, and the *t*-test was used for the intergroup comparisons. We evaluated the collected data statistically and graphically using GraphPad Prism software (GraphPad Software Inc., San Diego, CA, United States).

## Results

### Anthropometric parameters of the participants in the obesity group and the healthy weight group

**FIGURE 1:**
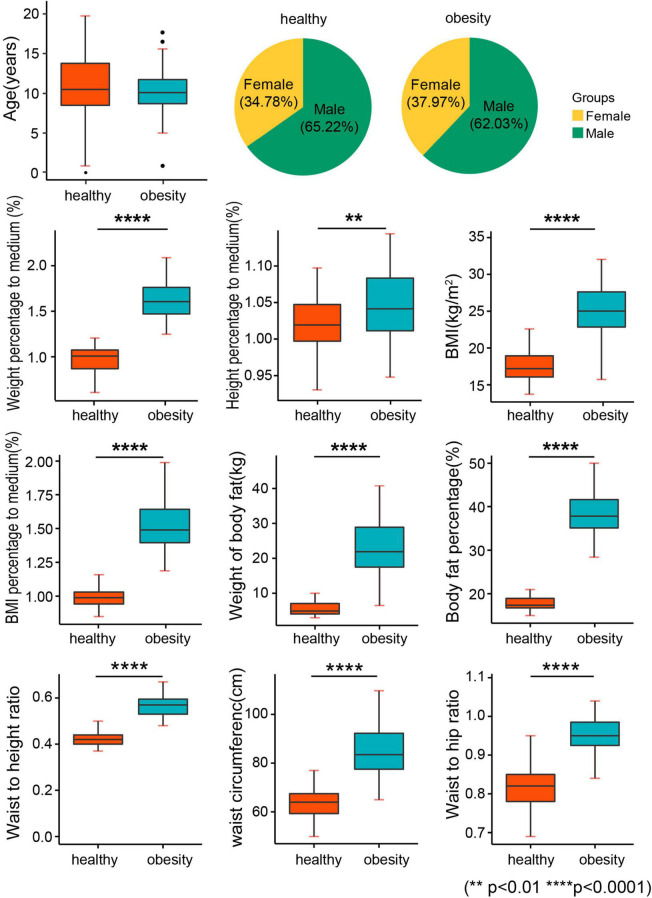
Baseline characteristics. BMI, body mass index; weight percentage to medium: the percentage of body weight to the median of standard body weight; height percentage to medium: the percentage of height to the median of standard height; BMI percentage to medium: the percentage of BMI to the median of standard BMI.

After randomized enrollment, 80 children with obesity and 161 children with normal weight were enrolled in the current study for investigation. One child with obesity was excluded due to the prescription of drugs for psychosis. Finally, 79 children with obesity and 161 normal children were included. The obesity group and the normal weight group were comparative on the age and sex ratio (Table 1 and Figure 1). However, a significant difference was found between the obesity group and the healthy weight group in the anthropometric parameters of obesity, such as weight percentage to medium, height percentage to medium, BMI adjusted by sex and age, BMI percentage to medium, waist circumference, hip circumference, WHR, and WHtR (Table 2 and Figure 1). Moreover, human component analysis showed that body fat content and BFP in the obesity group were significantly higher than those in the healthy weight group (38.1 vs. 17.7%) (Table 2 and Figure 1).

**TABLE 1:** Baseline characteristics.

	Healthy (*n* = 161)	Obesity (*n* = 79)	*P*
Age(y)	10.18 ± 2.56	10.87 ± 3.29	0.133
Male/Female	105/56 (65.2/34.8%)	49/30 (62.0/38.0%)	0.628
Gestation (week)	39.05 ± 1.00	38.81 ± 2.13	0.8075
Born weight (kg)	3.16 ± 0.40	3.25±0.57	0.6956
Born length (cm)	50.60 ± 2.88	50.30 ± 2.79	0.8263
Breastfeeding duration (month)	7.93 ± 7.25	6.00 ± 2.38	0.4962
Self-solid food introduction time (month)	5.57 ± 0.79	6.39 ± 6.04	0.7248
Dentition time (month)	7.93 ± 1.97	6.44 ± 2.13	0.1100
Sleep time (h/d)	8.86 ± 0.75	8.79 ± 0.85	0.8441

**TABLE 2:** Anthropometric parameters.

	Healthy (*n* = 161)	Obesity (*n* = 79)	*P*
Recent weight (kg)	39.76 ± 12.68	55.44 ± 17.16	0.000***
Recent height (cm)	149.21 ± 15.24	146.64 ± 16.61	0.294
BMI (kg/m^2^)	17.74 ± 2.42	25.40 ± 3.47	<0.0001***
Weight percentage to medium	0.97 ± 0.17	1.61 ± 0.31	<0.0001***
height percentage to medium	1.02 ± 0.04	1.042 ± 0.05	0.0007***
BMI percentage to medium	1.00 ± 0.08	1.51 ± 0.18	<0.0001***
Waist circumference (cm)	63.97 ± 7.11	85.76 ± 10.63	<0.0001***
Waist to hip ratio (WHR)	0.82 ± 0.05	0.95 ± 0.05	<0.0001***
Waist to height ratio (WHtR)	0.43 ± 0.04	0.51 ± 0.20	0.0052**
Weight of body fat (kg)	5.49 ± 2.25	23.00 ± 8.83	<0.0001***
Body fat percentage (BFP, %)	17.72 ± 1.58	38.09 ± 5.32	0.000***

**P < 0.01, ***P < 0.001.

### Potential factors associated with the incidence of childhood obesity

#### Basic data related to birth, family, and living habits of participants

We collected basic data related to birth weight, birth length (Table 1), and other baseline characteristics (e.g., parents’ BMI and living area) (Table 3). We found that the participants in the healthy weight group lived in higher floors than the obesity group (*p* = 0.0437), which indicated that climbing the stairs may be an effective method to decrease the incidence of childhood obesity.

**TABLE 3:** Parents’ stature and family environment.

	Healthy (*n* = 40)	Obesity (*n* = 63)	*P*
Father weight (kg)	84.67 ± 12.68	72.87 ± 10.75	0.4210
Father BMI	23.58 ± 2.29	25.05 ± 3.77	0.3670
Mother weight (kg)	53.57 ± 2.44	58.41 ± 10.22	0.2290
Mother BMI	21.73 ± 1.43	22.87 ± 3.18	0.4024
Floor height	4.50 ± 0.65	2.29 ± 0.61	0.0437*
Floor area	91.29 ± 42.41	142.00 ± 79.72	0.1218

*P < 0.05.

#### Dietary behaviors

**FIGURE 2:**
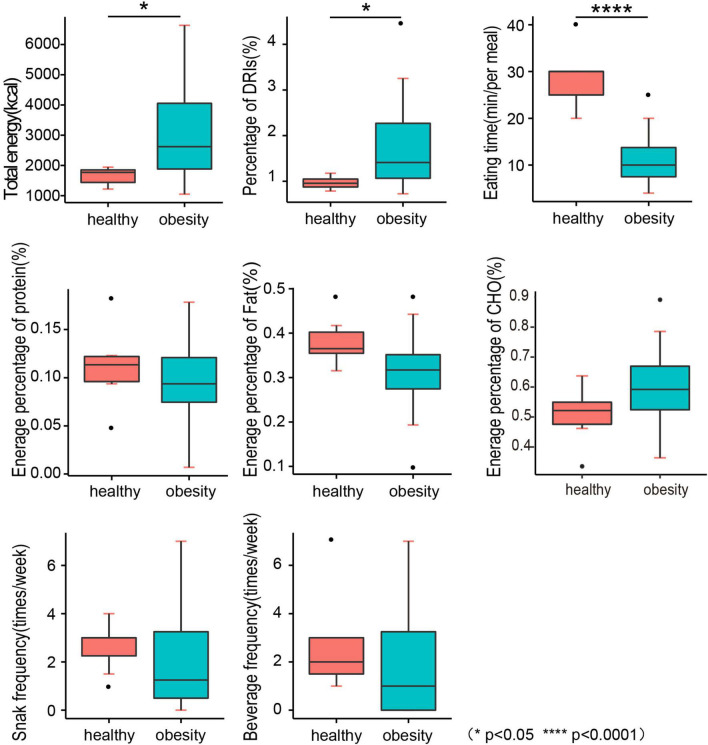
Dietary survey and dietary behavior. DRIs, dietary reference intakes; CHO, carbohydrate.

We conducted 24-h recall and a 3-day dietary survey to assess dietary behaviors. We found that children with obesity significantly ate faster than the normal weight children (*p* < 0.0001) (Figure 2 and Table 4), and a higher total energy intake was noted in children with obesity (*p* = 0.0234) compared with the DRIs (*p* = 0.0395) (Figure 2 and Table 4). This dietary behavior might cause eating more, and it was strongly associated with the occurrence of childhood obesity in Guangzhou. No significant differences were found between the two groups in terms of the percentage of energy from fat, carbohydrates, proteins, snacks, as well as beverage frequency.

**TABLE 4:** Dietary survey and dietary behavior.

	Healthy (*n* = 40)	Obesity (*n* = 63)	*P*
Total energy (kcal)	1646 ± 265.63	2995 ± 1248.06	0.0234*
Percentage to DRI (%)	96.63 ± 14.00	171.25 ± 87.12	0.0395*
Energy percentage of CHO (%)	50.63 ± 3.55	58.83 ± 2.42	0.1095
Energy percentage of FAT (%)	38.22 ± 2.05	31.56 ± 1.73	0.0634
Energy percentage of PRO (%)	11.14 ± 1.54	9.61 ± 0.80	0.3742
Eating time (min/per meal)	28.57 ± 2.61	11.37 ± 1.33	<0.0001***
Snack frequency (times/week)	2.64 ± 0.39	2.27 ± 0.54	0.5832
Beverage frequency (times/week)	2.71 ± 0.78	2.14 ± 0.55	0.6007

*P < 0.05, ***P < 0.001.

### Blood lipid profile and potential risk factors in cardiovascular disease

**FIGURE 3:**
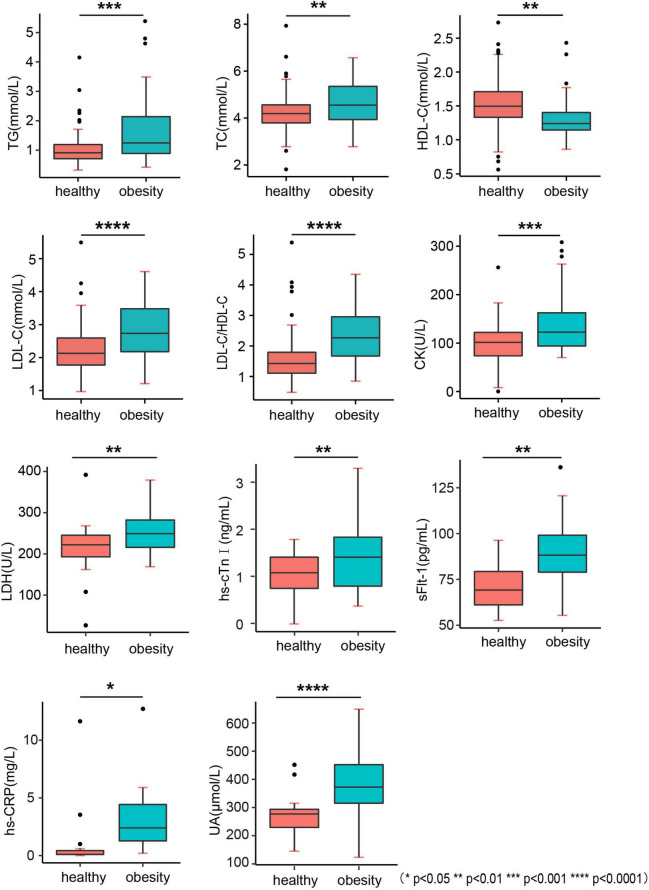
Biochemistry indicators. TG, triglyceride; TC, total cholesterol; HDL-C, high-density cholesterol; LDL-C, low-density lipoprotein cholesterol; LDL-C/HDL-C, LDL-C to HDL-C ratio; CK, creatine kinase; LHD, lactate dehydrogenase; hs-cTnI, high-sensitivity cardiac troponin I; sFlt-1, fms-like tyrosine kinase-1; hs-CRP, high-sensitivity C-reactive protein; UA, uric acid.

Children in the obesity group had an abnormal blood lipid profile in terms of triglyceride (TG), total cholesterol (TC), high-density cholesterol (HDL-C), low-density lipoprotein cholesterol (LDL-C), and LDL-C to the HDL-C ratio (LDL-C/HDL-C) compared with those in the healthy weight group (Figure 3). However, no significant differences were found in other parameters, such as apolipoprotein A1 (ApoA1), apolipoprotein B (ApoB), and non-esterified fatty acids (NEFA) (Table 5).

**TABLE 5:** Biochemistry indicator and cardiovascular risk factors.

	Healthy (*n* = 157)	Obesity (*n* = 69)	*P*
TG (mmol/L)	1.01 ± 0.04	1.68 ± 0.16	0.0001***
TC (mmol/L)	4.22 ± 0.06	4.62 ± 0.13	0.0070**
HDL-C (mmol/L)	1.54 ± 0.03	1.31 ± 0.04	<0.0001***
LDL-C (mmol/L)	2.22 ± 0.06	2.88 ± 0.12	<0.0001***
LDL-C/HDL-C	1.54 ± 0.06	2.31 ± 0.12	<0.0001***
ApoA1 (g/L)	1.12 ± 0.07	1.14 ± 0.03	0.8925
ApoB (g/L)	0.81 ± 0.06	0.88 ± 0.04	0.2940
ApoE (g/L)	37.11 ± 6.85	44.65 ± 2.83	0.2339
NEFA (mmol/L)	0.47 ± 0.06	0.48 ± 0.06	0.8904
CK (U/L)	100.00 ± 8.96	151.50 ± 15.97	0.0062**
CK-MB (U/L)	24.53 ± 3.60	17.15 ± 1.38	0.0622
LDH (U/L)	214.30 ± 9.72	250.90 ± 6.11	0.0012**
NT-proBNP (pg/ml)	36.93 ± 23.30	28.74 ± 11.86	0.1353
Myoglobin (ng/ml)	23.28 ± 1.83	25.12 ± 2.53	0.5605
hs-cTnI (pg/ml)	1.09 ± 0.13	1.48 ± 0.16	0.0042**
sFlt-1 (pg/ml)	70.69 ± 3.03	89.91 ± 4.39	0.0013**
IL-6 (pg/ml)	7.31 ± 4.35	35.50 ± 30.86	0.3765
hs-CRP (mg/L)	1.042 ± 0.66	3.09 ± 0.55	0.0225*
UA (μmol/L)	273.80 ± 15.82	391.10 ± 15.12	<0.0001***

*P < 0.05, **P < 0.01, ***P < 0.001.

The levels of oxidation indices, such as high-sensitivity C-reactive protein (hs-CRP) and uric acid (UA), were higher in the obesity group, indicating the low-grade inflammation in children with obesity (Figure 3 and Table 5). The levels of biomarkers of myocardial injury, including creatine kinase (CK), lactate dehydrogenase (LHD), high-sensitivity cardiac troponin I (hs-cTnI), and soluble fms-like tyrosine kinase-1 (sFlt-1) (*p* = 0.0062, 0.0012, and 0.0013, respectively) were also higher in the obesity group, indicating the potential of myocardial damage and fibrosis in the heart of children with obesity (Figure 3 and Table 5).

### Blood pressure and cardiac dysfunction in the obesity group

**FIGURE 4:**
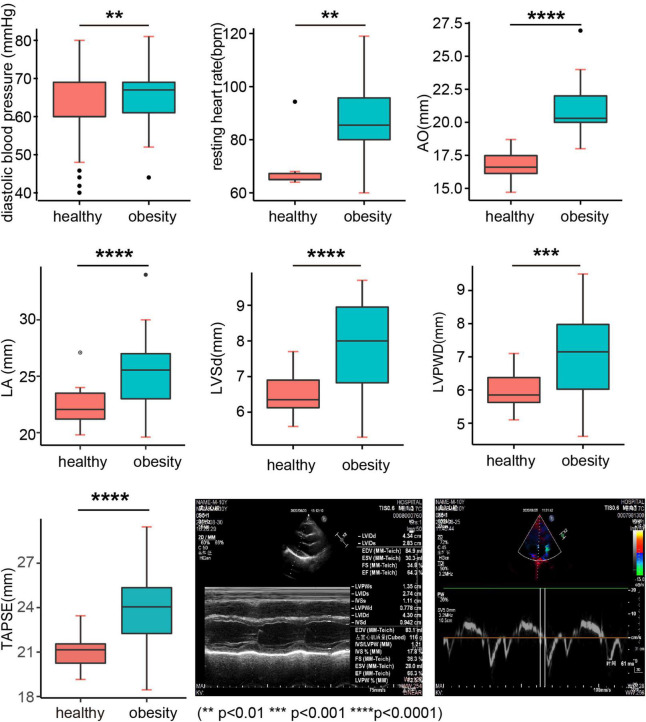
Cardiovascular functional parameters. AO, aortic annulus; LA, left atrial diameter; IVSd, interventricular septal wall thickness at end diastole; LVPWD, left ventricular posterior wall thickness at end diastole; TAPSE, tricuspid annular plane systolic excursion.

**FIGURE 5:**
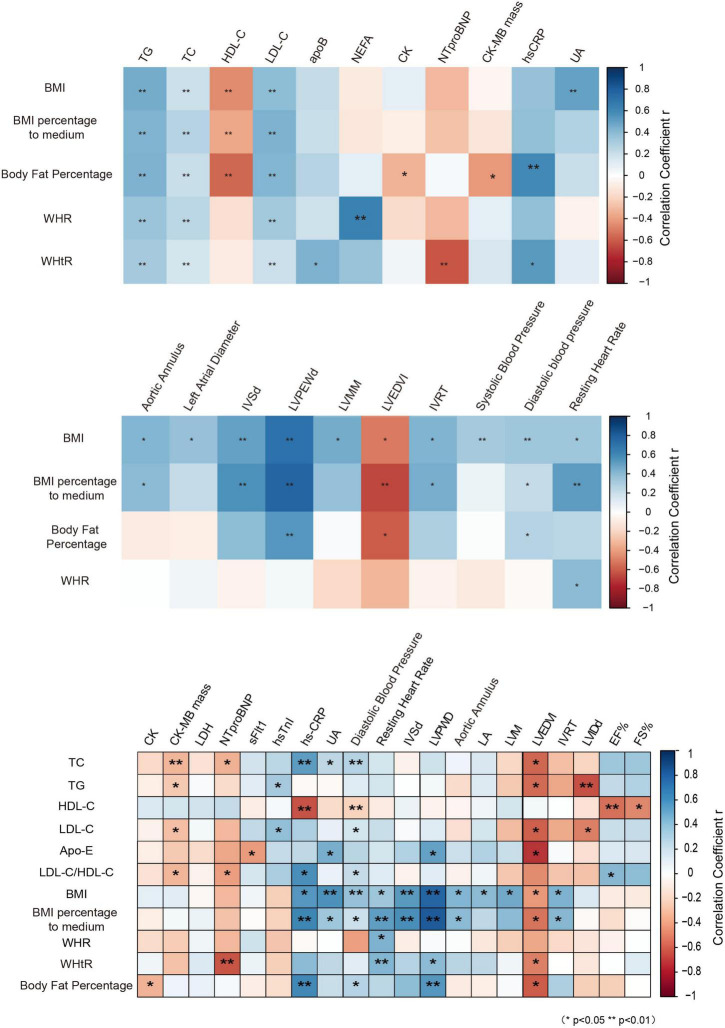
Correlation analysis of obesity indicators with cardiovascular risk factors. BMI, body mass index; WHR, waist-to-hip ratio; WHtR, waist-to-height ratio; NEFA, non-esterified fatty acids; apoB, apolipoprotein B; NTproBNP, N-terminal pro-brain natriuretic peptide; CK-MB mass, creatine kinase-MB mass; AO, aortic annulus; LA, left atrial diameter; IVSd, interventricular septal wall thickness at end diastole; LVPWD, left ventricular posterior wall thickness at end diastole; LVM, left ventricular mass; LVMM, left ventricle myocardial mass; LVEDVI, left ventricular end-diastolic volume index; IVRT, isovolumetric relaxation time; LVIDd, end-diastolic left ventricular internal diameter; EF%, left ventricular ejection fraction; FS%, fractional shortening.

As shown in Table 6, systolic blood pressure (SBP) was not significantly raised in the obesity group; however, diastolic blood pressure (DBP) and the resting heart rate were significantly higher than those in the healthy weight group (Figure 4). Color Doppler echocardiography further showed that left and/or right ventricular functions were attenuated in 52.4% of children with obesity. In particular, the values of some functional parameters, such as AO, LA diameter, IVSd, LVPWD, and TAPSE, were significantly raised in the obesity group compared with those in the healthy weight group (Table 7 and Figure 5), suggesting that obesity status indicated the heart function before manifestation of clinical symptoms, and the cardiac dysfunction was typically characterized by diastolic dysfunction (Figure 4 and Table 7).

**TABLE 6:** Blood pressure.

	Healthy (*n* = 161)	Obesity (*n* = 79)	*P*
Systolic blood pressure Systolic BP (mm/Hg)	103.50 ± 0.87	103.80 ± 1.62	0.8712
Diastolic blood pressure Diastolic BP (mm/Hg)	61.94 ± 0.71	65.79 ± 1.14	0.0074**
Resting heart rate (bpm)	70.17 ± 4.80	87.33 ± 2.08	0.0049**

**P < 0.01.

**TABLE 7:** Left and right ventricular function parameters.

Measured	Reference (*n* = 40)	Obesity (*n* = 40)	*P*
Aortic annulus (AO, mm)	16.72 ± 0.92	21.02 ± 1.94	0.000***
Left atrial diameter (LA, mm)	22.30 ± 1.50	25.22 ± 3.19	0.000***
End-diastolic left ventricular internal diameter (LVIDd, mm)	42.48 ± 1.80	43.75 ± 8.52	0.1560
End-systolic left ventricular internal (LVIDs, mm)	26.36 ± 1.11	27.09 ± 3.20	0.2433
Interventricular septum at end-diastole (IVSd, mm)	6.49 ± 0.51	7.77 ± 1.30	0.000***
Left ventricular posterior wall thickness at end diastole (LVPWD, mm)	5.97 ± 0.48	6.97 ± 1.36	0.0003***
Fractional shortening (FS%)	37.94 ± 1.08	37.85 ± 3.15	0.5204
Left ventricular ejection fraction (EF%)	66.58 ± 0.83	68.66 ± 4.18	0.0841
Left ventricle myocardial mass (LVMM, g)	37.39 ± 0.40	37.85 ± 0.64	0.6315
Tricuspid annular plane systolic excursion (TAPSE, mm)	20.76 ± 0.16	23.67 ± 0.52	<0.0001***
Right ventricular diameter (RVD, mm)	19.37 ± 1.10	18.87 ± 2.25	0.2845

***P < 0.001.

AO, aortic annulus; LA, left atrial diameter; LVIDd, end-diastolic left ventricular internal diameter; LVIDs, end-systolic left ventricular internal; IVSd, interventricular septal wall thickness at end diastole; LVPWD, left ventricular posterior wall thickness at end diastole; FS%, fractional shortening; EF%, left ventricular ejection fraction; RVD, right ventricular diameter; LVMM, left ventricle myocardial mass; TAPSE, tricuspid annular plane systolic excursion; RVD, right ventricular diameter.

### Correlation analysis of obesity indicators with cardiovascular risk factors

Biomarkers of cardiovascular disease are clinically valuable to predict the potential risk of cardiovascular disease. However, they are costly and time-consuming. The association between the blood lipid profile and biomarkers of cardiovascular disease was widely studied in adults, so that the blood lipid profile can be a potentially alternative marker to predict the risk of cardiovascular events. However, whether it could predict cardiac dysfunction in children with obesity has still remained elusive.

Using correlation analysis, it was found that the anthropometric parameters of obesity, such as BMI, BFP, and waist circumference, were highly correlated with the blood lipid profile, and with some biomarkers of cardiovascular disease, and most of the parameters for evaluation of cardiac function. Furthermore, it was revealed that the anthropometric parameters of obesity, such as BMI, percentage of BMI to medium, WHtR, and BFP, were significantly positively correlated with most of the parameters for evaluation of cardiac function than the blood lipid profile (Figure 5), suggesting that the anthropometric parameters of obesity may be economically alternative biomarkers for cardiac dysfunction in children with obesity in Guangzhou.

## Discussion

In current study, we investigated the cardiovascular features of obese children in Guangzhou, and found that obesity status affected the cardiac function that was typically characterized by diastolic dysfunction, and the anthropometric parameters of obesity could serve as economically alternative biomarkers for cardiac dysfunction in obesity children.

Obesity is a major public health concern that is associated with the development of cardiovascular risk factors during childhood and adolescence, as well as premature mortality in adults (13). However, the association of obesity with an abnormal cardiac function still needs to be deeply investigated. A study found that the left ventricular (LV) mass/height increased in the obesity group, and obesity children had regional longitudinal peak systolic myocardial deformation properties lower than those of referents in both the left and right ventricles (14). A systematic review and meta-analysis suggested that childhood obesity is significantly and positively associated with adulthood SBP, DBP, and TG, while it significantly and inversely correlated with adulthood HDL-C (13). In our study, we found higher incidence rates of hyperlipidemia and hypertension in the obesity group; however, we did not observe the significant increase of SBP in the obesity group. Regarding cardiac function assayed by the echocardiography, LA diameter, IVSd, LVPWD, and AO increased in the obesity group compared with those in the healthy weight group, and the left and/or right ventricular functions were declined in 52.4% of obese children. Some studies demonstrated that elevated SBP occurs more frequently in men than in women, and boys are more susceptible to develop high SBP as they approach adulthood compared with girls (15, 16). We, in this study, did not compare SBP between boys and girls, as no gender effect on SBP of obese patients was assessed, particularly due to the limited sample size. Even in the absence of hypertension, the increased total blood volume in obese individual’s increases stroke volume, while total peripheral resistance decreases and the heart rate may be normal or slightly elevated (17). It is noteworthy that patients with obesity and diabetes exhibited high prevalence of cardiac diastolic dysfunction as an independent predictor of cardiovascular events for which no evidence-based treatment exists (18). In contrast to systolic function, studies found that subclinical left ventricular diastolic dysfunction is present in all grades of isolated obesity. Cardiac diastolic function is important for the maintenance of normal function and mainly decreases earlier than the systolic function with aging (19). In our study, the increase of DBP and the resting heart rate indicated the reduction of cardiac function. Thus, early warning and preventive measures are of great significance for childhood obesity.

Cardiac dysfunction in obese children was also confirmed by the increased blood biomarkers for heart failure, such as hs-cTnI and sFlt-1. Both oxidative stress and inflammation are involved in the pathogenesis of cardiovascular disease (20). We found the significant increase of inflammation and oxidative stress markers (e.g., hs-CRP and UA) in obese children, which were consistent with previously reported findings (21). Thus, we analyzed the relationship between obesity and these cardiovascular indicators, indicating that obesity affected cardiovascular function, including cardiac structures confirmed by Color Doppler echocardiography, indicating the necessity of preventing cardiac damage by controlling obesity.

Exploration of an effective method to monitor the potential effects on cardiovascular system in obese children is of great significance for the prevention of long-term damage. Hypertriglyceridemia, resulting from either the increased TG production or the reduced TG clearance, is highly prevalent in children and adolescents with obesity. A genetic study suggested that TG and TG-rich lipoproteins and, more specifically, remnant cholesterol are in the causal pathway of cardiovascular disease (22). The TG/high-density lipoprotein cholesterol (HDL-C) ratio was reported as a marker of structural vascular changes and insulin resistance in obese youth (23). Measurement of lipid profiles in obese children and overweight adolescents is a useful marker for estimating the potential risk of cardiovascular disease (23). Lactate dehydrogenase (LDH) isoenzymes can be used as valuable diagnostic markers for metabolic syndrome. This may assist scholars to explore the metabolic changes associated with obesity and diabetic complications (24). Overweight and obesity are associated with elevated biochemical markers of liver damage. The elevated aspartate transaminase (AST) may reflect muscle, heart, or pancreas injury, and LDH is, generally, a marker of hemolysis and tissue breakdown. LDH may be a marker of obesity-related liver injury (25).

In a study conducted by Sudhir et al. (26), children with elevated BMI with normal waist circumference (NWC) had an LVM profile similar to children with normal BMI with NWC, indicating the importance of association between waist circumference and LVM. The correlation analysis of anthropometric parameters with the blood lipid profile showed that the anthropometric parameters were strongly associated with the lipid profiles and had a more specific correlation with most of cardiac dysfunction-related parameters in obese children, suggesting that anthropometric parameters may serve as alternative markers for cardiovascular risk factors, especially for cardiac dysfunction. The results of the current study may assist scholars to develop relatively accurate screening models for cardiovascular risk factors in obese children, and to avoid utilization of costly biomarkers for cardiac function tests.

Lifestyle changes and dietary interventions play important roles in obesity-associated hypertriglyceridemia. Dietary restriction of fat remains the mainstay of management of primary hypertriglyceridemia (22). Our results and some previous studies have shown that hypertriglyceridemia may be associated with some cardiovascular risk factors (27, 28); however, they may not be well-correlated with cardiac dysfunction. The strong correlation between anthropometric parameters and cardiac dysfunction makes the anthropometric parameters as an early warning signal and a correct biomarker for clinical intervention, and changes in anthropometric parameters through specific measures can effectively reduce the occurrence of heart injury in obese children.

Adipose tissue (AT) would serve as a secretory organ (29). Different adipose depots have also been associated with distinct adipokine secretion patterns, comprising leptin and Adiponectin (30). In addition, adipose depots exhibit different inflammatory and secretory profiles that may contribute to distinct cardiometabolic risk. A-FABP, adipocyte fatty acid–binding protein; IL-6, interleukin-6, IL-1b, interleukin-1b; sTNFR2, soluble tumor necrosis factor receptor 2; TNF-a, tumor necrosis factor-a.

Chronic heart failure (HF) is characterized by a neurohormonal dysfunction associated with chronic inflammation. Adiponectin, an adipose-tissue-derived cytokine, seems to play an important role in cardiac dysfunction (31). Some research data confirm the rise of adiponectin in overt HF. The levels of circulating adipokine seem to be mainly predicted by the metabolic profile of patients and by biohumoral indicators, rather than by clinical and echocardiographic indexes of HF severity.

Independent of the underlying pathophysiology, cardiac tissue damage induces an inflammatory response to initiate repair processes. Immune cells are recruited to the heart to remove dead cardiomyocytes, which is essential for cardiac healing. Insufficient clearance of dying cardiomyocytes after myocardial infarction (MI) has been shown to promote unfavorable cardiac remodeling, which may result in HF. Although immune cells are integral key players of cardiac healing, an unbalanced or unresolved immune reaction aggravates tissue damage that triggers maladaptive remodeling and HF (32). Therapeutic manipulation of systemic metabolism can reestablish immune cell homeostasis and consequently modulate adverse cardiac remodeling. This might be achieved *via* nutrition therapy (33) or *via* metabolic drugs to improve homeostasis of metabolic organs (liver, adipose tissue, and pancreas). However, maladaptive immune cell behavior contributes to ventricular remodeling in mouse models, prompting experimental efforts to modulate the immune response to prevent the development of HF. However, maladaptive immune cell behavior contributes to ventricular remodeling in mouse models, prompting experimental efforts to modulate the immune response to prevent the development of HF (34).

Hypertriglyceridemia obviously exists among obese children; however, the limitations of the lipid biomarker have been found in recent studies. Deferent from increased Triglycerides in 6 h after administration of a standardized oral fat load (OFL) to fasting patients (35), a study found that, in overweight male subjects with the metabolic syndrome, an oral fat load is accompanied with a modest anti-inflammatory response of adipose tissue-derived adipocytokines, e.g., Adiponectin (36). A Mendelian Randomization study demonstrates that LDL cholesterol and triglycerides are associated with adverse changes in cardiac structure and function, in particular in relation to LV mass. These findings suggest that LDL cholesterol and triglycerides may have a causal effect on influencing cardiac morphology in addition to their established role in atherosclerosis (37). Among the lipoproteins, some researchers found serum apo B was significantly and inversely associated with LV dilatation, independently of conventional lipids and other CV risk factors in patients with end-stage renal disease undergoing peritoneal dialysis. It suggested that the low serum apo B level could be a powerful risk marker for eccentric left ventricular geometry remodeling (38).

A relatively small sample size is one of the limitations of our study. Moreover, no data related to the effects of dietary interventions on obese children’ quality of life were collected.

## Conclusion

In summary, the present study suggested that obese children in Guangzhou suffered from functional damages related to cardiovascular events, which were characterized by cardiac diastolic dysfunction, and the anthropometric parameters of obesity could be economically alternative biomarkers for monitoring of cardiac dysfunction in obese children.

## Data availability statement

The original contributions presented in the study are included in the article/supplementary material, further inquiries can be directed to the corresponding authors.

## Ethics statement

The studies involving human participants were reviewed and approved by the Ethics Committee of Guangzhou Women and Children’s Medical Center (number 375B00). Written informed consent to participate in this study was provided by the participants’ legal guardian/next of kin. Written informed consent was obtained from the individual(s) and minor(s)’ legal guardian/next of kin, for the publication of any potentially identifiable images or data included in this article.

## Author contributions

JS and LW performed the data analysis with the assistance of YJL, YFL, FL, WC, and WD. XML performed echocardiography and data were interpreted by HC, MX, and HL. JS, XHL, and JD designed the study and prepared the manuscript. All authors contributed to the article and approved the submitted version.
